# The Efficacy of Endoscopic Submucosal Dissection of Type I Gastric Carcinoid Tumors Compared with Conventional Endoscopic Mucosal Resection

**DOI:** 10.1155/2014/253860

**Published:** 2014-02-17

**Authors:** Hyung Hun Kim, Gwang Ha Kim, Ji Hyun Kim, Myung-Gyu Choi, Geun Am Song, Sung Eun Kim

**Affiliations:** ^1^Department of Internal Medicine, The Catholic University of Korea College of Medicine, Seoul 137-701, Republic of Korea; ^2^Department of Internal Medicine, Pusan National University, School of Medicine, Busan 614-735, Republic of Korea; ^3^Department of Internal Medicine, Inje University College of Medicine, Busan Paik Hospital, Gaegeum-dong, Busanjin-Gu, Busan 602-739, Republic of Korea; ^4^Department of Internal Medicine, Kosin University College of Medicine, Busan 602-702, Republic of Korea

## Abstract

*Background and Aims*. Conventional endoscopic submucosal resection (EMR) of carcinoid tumors often involves the resection margin, which necessitates further intervention. Endoscopic submucosal dissection (ESD) is widely accepted for removing carcinoid tumors. We aimed to evaluate the clinical usefulness of ESD with that of EMR for resection of type I gastric carcinoid tumors. *Patients and Methods*. The study enrolled 62 patients (37 males, 25 females; median age, 50 years; range, 40–68 years) who were treated with EMR or ESD at three hospitals; the study group had 87 type I gastric carcinoid tumors with an estimated size of ≤10 mm. The complete resection rate and the complications associated with these two procedures were analyzed. *Results*. The overall ESD complete resection rate was higher than that of the EMR rate (94.9% versus 83.3%, *P* value = 0.174). A statistically lower vertical margin involvement rate was achieved when ESD was performed compared to when EMR was performed (2.6% versus 16.7%, *P* value = 0.038). The complication rate was not significantly different between the two groups. *Conclusions*. ESD showed a higher complete resection rate, particularly for the vertical margin, with a similar complication rate. We mildly recommend ESD rather than EMR for removing type I gastric carcinoid tumors.

## 1. Introduction

Although being rare, the incidence of gastric carcinoid tumors has significantly increased over the past 50 years. The percentage of gastric carcinoid tumors among all gastric malignancies has increased from 0.3 to 1.77% since the 1950s, and the proportion of gastric carcinoid tumors among all gastrointestinal carcinoid tumors has increased from 2.4 to 8.7% [1]. Undoubtedly, increased endoscopic surveillance and enhanced evaluation of gastric biopsies are partially responsible for this observed increase [2]. As knowledge of the pathogenesis and clinical significance of gastric carcinoid tumors evolves, clinicians are better able to identify and manage these tumors [2]. Gastric carcinoid tumors are classified into three categories: type I, arising in atrophic body gastritis, type II, a manifestation of type I multiple endocrine neoplasia (MEN-I), and type III, with no specific background disease [3]. The type I variety comprises 70 to 80% of gastric carcinoid tumors. Type I gastric carcinoid tumors are often associated with chronic atrophic gastritis. Approximately 5% of patients with autoimmune chronic atrophic gastritis develop gastric carcinoid tumors [4–6]. Most type I gastric carcinoid tumors have a benign course, with metastasis occurring in <10% of tumors measuring <2 cm and in 20% of larger tumors [2]. Given this low potential for malignancy, the medical and surgical treatments are controversial. Even with no treatment, there is little risk for local or distant metastases, and the carcinoid tumors may even regress over time [7]. In general, most clinicians would agree that type I gastric carcinoid tumors that measure <1 cm are fewer than five in number, and with no lesions extending beyond the submucosa, endoscopic resection is the optimal treatment choice [2, 8–12]. Endoscopic submucosal dissection (ESD) has been approved for en bloc and complete resection of early gastric cancer, particularly in Korea and Japan, and it is considered to be the best technique for these lesions [13–15]. Moreover, this technique has been used to remove rectal carcinoid tumors and has demonstrated a superior efficacy to conventional endoscopic mucosal resection (EMR), particularly for less vertical margin involvement [16, 17]. However, few reports have evaluated the efficacy of ESD compared with EMR in type I gastric carcinoid tumors [18]. The aim of this study was to investigate whether ESD is superior to EMR for removing type I gastric carcinoid tumors as a means of complete resection.

## 2. Materials and Methods

### 2.1. Patients and Lesions

**Figure 1:**
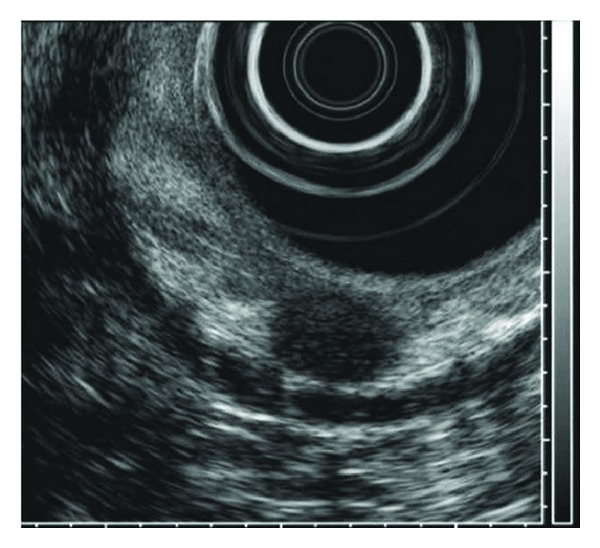
An approximately 1 cm × 0.8 cm, round, homogenous hypoechoic lesion located in the submucosal layer was detected using endoscopic ultrasonography.

Between January 2004 and December 2012, 62 patients (37 males, 25 females; median age, 50 years; range, 40–68 years) were treated with EMR or ESD at three hospitals; the study group had 87 type I gastric carcinoid tumors with an estimated size of ≤10 mm. The tumor size was estimated endoscopically with measuring forceps (Olympus Co., Tokyo, Japan). No patient had carcinoid syndrome symptoms. All tumors were found incidentally during a screening endoscopy. No tumor invasion beyond the submucosal layer was observed on the endoscopic ultrasonography before ESD or EMR (Figure 1). Computed tomography (CT) revealed that none of the tumors were associated with lymph node metastasis or with distant metastasis. All patients provided written informed consent for the endoscopic treatment of type I gastric carcinoid tumors. The patients' medical records were reviewed retrospectively to extract clinical information, including endoscopic findings (e.g., the tumor characteristics and type of surrounding gastric mucosal atrophy), laboratory findings, resection techniques, and any complications. This retrospective study was approved by the institutional review board of each participating institution: Kosin University College of Medicine, Inje University College of Medicine, and Pusan University College of Medicine.

### 2.2. ESD and EMR Procedures

**Figure 2:**
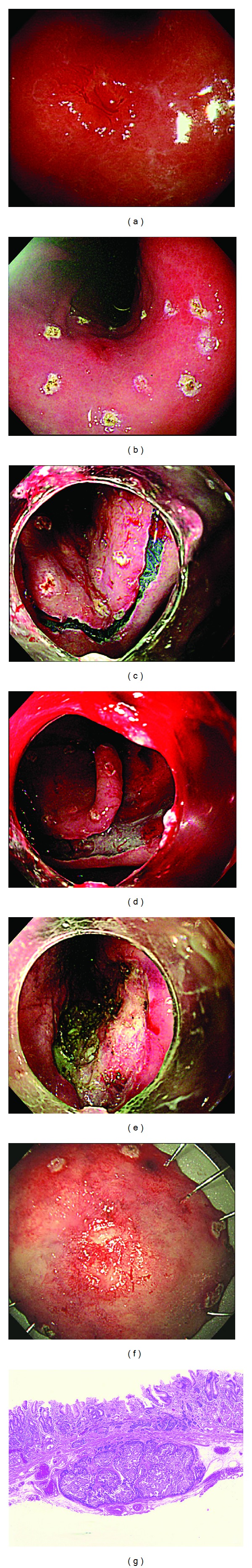
(a) An approximately 1 cm, round, slightly elevated subepithelial lesion with a central hyperemic depression was observed at the anterior wall side of the lower body. (b) The marking was performed using an argon plasma coagulator. (c) A circumferential mucosal incision was performed after a submucosal injection. (d) A submucosal dissection being performed. (e) A large artificial ulcer created by endoscopic submucosal dissection for a type I gastric carcinoid tumor was observed. (f) Endoscopic en bloc resection was achieved. (g) Histologic findings (hematoxylin and eosin (H&E) stain, ×40) showed complete resection: negative lateral and negative deep resection margin.

**Figure 3:**
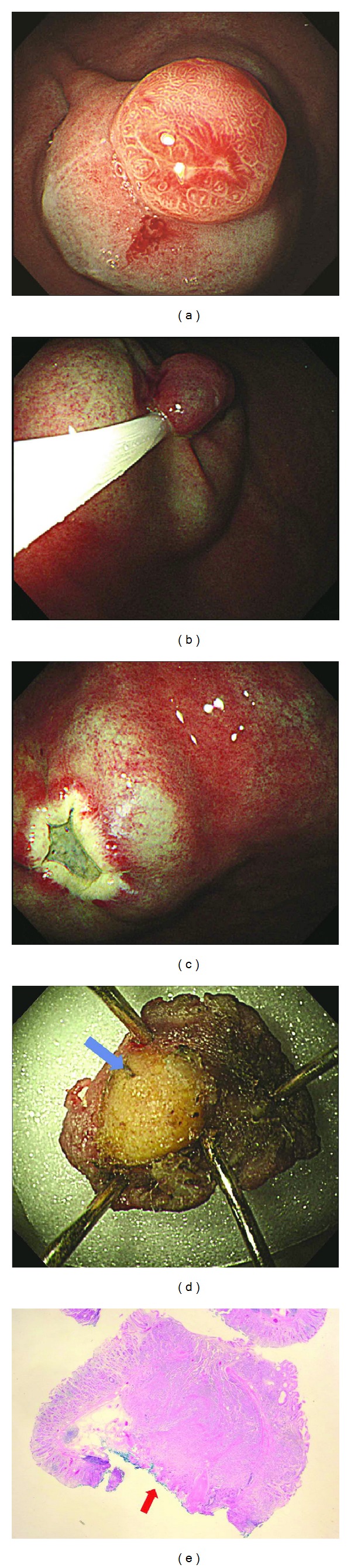
(a) A hyperemic polypoid subepithelial lesion measuring approximately 1 cm was detected at the greater curvature side of the upper body. (b) Submucosal injection was performed. After then, an endoscopic mucosal resection was performed using a snare. (c) A small artificial ulcer produced by endoscopic mucosal resection was detected. (d) Endoscopic en bloc resection was achieved, but a yellowish vertical resection margin that was not covered with submucosal tissue was manifested (blue arrow). (e) The histologic findings (H&E stain, ×10) showed a positive deep resection margin (red arrow).

All ESD practitioners are experts who performed over 300 cases of ESD. The ESD procedures were performed as described previously in detail with room air insufflations (Figure 2) [19]. After marking around the lesion, normal saline containing epinephrine was injected into the submucosal layer of the lesion. A circumferential mucosal incision around the lesion and submucosal dissection for the complete removal of the lesion were performed using various knives. To control bleeding during the procedure or to prevent possible bleeding from visible vessels in the artificial ulcer immediately after the resection, hemostatic forceps were used. EMR was performed, as follows (Figure 3). After a submucosal injection of saline with epinephrine, the open snare was placed around a portion of the lesion and was gently pressed against the mucosa. The excess air was aspirated to decrease the distension and facilitate grasping of the targeted lesion. After snare excision, the lumen was insufflated again to visualize the resection area. Hemostatic forceps were applied to ablate any possible bleeding foci in the EMR-related ulcers.

### 2.3. Evaluation of the Complete Resection and Complications

Endoscopic complete resection was defined as the en bloc resection of the lesion. All resected specimens were evaluated histologically using light microscopy at low-power and high-power magnifications. Histologic complete resection was defined as the lateral and vertical margin of the specimens (i.e., free of tumor invasion). Procedure-related bleeding was classified as immediate or delayed. Immediate bleeding was defined as bleeding during the procedure that did not cease spontaneously and that required hemostatic intervention, such as argon plasma/hemostatic forceps coagulation or clips (Easy clip, Olympus Co., Tokyo, Japan). Delayed bleeding was defined as the occurrence of hematemesis, hematochezia, or melena (after completing ESD or EMR) that required endoscopic and/or angiographic hemostasis or transfusions. Perforation was defined as gastric wall penetration recognized during the endoscopic procedure or detected after procedure via a radiological examination, such as X-ray or CT.

### 2.4. Statistical Analyses

All statistical analyses were performed using the Statistical Package for the Social Sciences (SPSS) software (version 16.0, SPSS, Chicago, IL, USA). Significant between-group differences were tested using Fisher's exact test and Student's *t-*test. *P* values for two-tailed tests were considered to be significant if they were <0.05.

## 3. Results

### 3.1. Patients and Tumors

Among the 87 lesions, 48 were resected using EMR and 39 were resected using ESD. No evidence of metastasis to the lymph nodes or other organs was found in the initial evaluation of any patient. The mean tumor size was  7.6 ± 4.1 mm, and according to the Kimura-Takemoto classification, open type atrophic gastritis was observed in 75.8% of the lesions. Tumors in the stomach body were more common (89%). There were no significant differences in baseline characteristics between the EMR and ESD groups, including age and gender (Table 1).

### 3.2. Endoscopic and Histologic Complete Resection Rates

The endoscopic complete resection rate was higher in the ESD group (38 lesions, 97.4%) than in the EMR group (45 lesions, 93.7%), but this difference was not statistically significant (*P*  value = 0.624). The histologic complete resection rate was much higher in the ESD group (37 lesions, 94.9%) than it was in the EMR group (40 lesions, 83.3%; *P*  value = 0.174). Involvement of the lateral resection margin was found in one case in the ESD group (2.6%) and in two cases in the EMR group (4.2%; *P*  value > 0.999). The rate of vertical resection margin involvement was significantly lower in the ESD group (one case, 2.6%) than it was in the EMR group (eight cases, 16.7%; *P*  value = 0.038; Table 2). The complete resection rate of the vertical margin achieved using ESD was 7.6-fold greater than that for EMR.

### 3.3. Microscopic Findings

Lymphatic invasion was observed in 1 case (1.1%); the patient underwent surgery regardless of histologic complete resection. No vascular invasion was identified in any patient. In no case did the tumor invade the muscular propria. The mitotic index findings were as follows: <2/high power field (HPF) 83 cases (95.4%) and 2–20/HPF, four cases (4.5%). In measuring the Ki 67 index, 72 cases (82.8%) showed <2% expression and 15 cases (17.2%) presented 3–20% expression. No difference was observed between the EMR and ESD groups (Table 3).

### 3.4. Complications and Procedure Duration

There were three immediate bleedings in the EMR group and six immediate bleedings in the ESD group that occurred during the procedures (*P*  value = 0.288). Two cases of delayed bleeding occurred eight hours after the procedure in both the ESD and EMR groups, respectively (*P*  value > 0.999). All bleeding cases were controlled endoscopically using clips and/or hemostatic forceps, and blood transfusions were not necessary. One perforation occurred in the ESD group; it was managed successfully by clipping. The procedure duration is much longer in ESD group (26.1 ± 10.5 min versus 9.5 ± 3.6 min, *P*  value < 0.001, Table 4).

## 4. Discussion

The present study found that ESD resulted in histologically complete resection in 94.9% of patients with type I gastric carcinoid tumors; this result was greater than the 83.3% rate achieved by using EMR. Although there were no significant differences in the overall complete resection rate and lateral margin involvement, a significantly lower vertical resection margin involvement was observed.

The higher histologically complete resection rate associated with ESD is clinically significant given the advantage of complete histological resection of type I gastric carcinoid tumors. First, complete resection is essential for determining the subsequent management options. Although the risk of metastasis from small type I gastric carcinoid tumors is generally low, some patients, even those with carcinoid tumors measuring <10 mm in diameter, may have metastases [20]. One recent study reported that 3.4% of tumors measuring <1 cm invading into the lamina propria or submucosa had lymph node metastases [20]. This observation demonstrates that it is safe to recommend additional resection with lymph node dissection if the endoscopically resected gastric carcinoid tumor specimens show lymphovascular invasion. To understand lymphovascular invasion accurately, it is necessary to acquire a substantial amount of submucosal tissue. For this reason, ESD is the better option compared with EMR.

A second advantage provided by complete histologic resection is that frequent endoscopic follow-up may not be required. Although a consensus protocol has yet to be established, it is recommended that follow-up endoscopy should not be delayed for too long after an incomplete gastric carcinoid tumor resection. Incomplete resection inevitably requires additional medical resources and inconveniences the patient.

Finally, a repeat of endoscopic resection of a remnant tumor after an initial incomplete endoscopic resection may be difficult (and even dangerous) because fibrosis prevents lifting the lesion using submucosal injection [21, 22]. Therefore, it would be reasonable to recommend acquiring histologically complete resection of type I gastric carcinoid tumors in the first trial when the lesions are small. This study showed that ESD may produce the most desirable patient outcomes.

In the ESD group, there were two instances of delayed bleeding and one case of perforation. The complication rate in the ESD group was similar to that in the EMR group and was similar to the rates that have been described in previous reports [23–25]. Therefore, we concluded that ESD can be a useful strategy for managing type I gastric carcinoid tumors based on the risk of complications.

The present study had several limitations. First, a retrospective study was used; a thorough review of available information was performed from the medical records. Therefore, we were unable to describe some intricate details about the procedures. However, we are certain that important data, including the resection rate and complications, were included. Second, some specific aspects of the ESD and EMR procedures might be different because we collected information from three different university hospitals, but this limitation was an inevitable part of the process to evaluate enough cases to produce a substantial statistical power, which was not acquired in the previous studies [18, 23–25]. Alternatively, this multicenter study has the advantage of producing data that can be generalized across centers. Third, the follow-up result for recurrence should be considered to adequately evaluate the efficacy of the practice. However, many patients were lost to follow up; therefore differences in recurrence rates with each technique could not be determined.

In conclusion, ESD need to be considered when treating type I gastric carcinoid tumors for the following two reasons despite of longer procedure duration compared with EMR. First, ESD yielded a significantly higher histologic complete resection rate than EMR, particularly in the vertical resection margin, resulting in complete histologic evaluation, less frequent endoscopic follow-up, and the prevention of additional endoscopic procedures. Second, the complication rates of ESD and EMR were similar. The long-term results after ESD and EMR treatments should be addressed in future prospective studies.

## Figures and Tables

**Table 1:** Baseline characteristics of patients with EMR and ESD groups.

	EMR (*n* = 48)	ESD (*n* = 39)	*P* value
Age, mean ± SD, year	53.2 ± 10.5	55.0 ± 10.2	0.626
Male, *n* (%)	18 (37.5)	22 (56.4)	0.427
Dyspepsia, *n* (%)	12 (25.0)	9 (23.1)	0.752
Endoscopic findings			
Open type atrophic gastritis,^†^ *n* (%)	38 (79.1)	28 (71.8)	0.459
Size of the tumor, mean ± SD, mm	7.8 ± 3.2	7.7 ± 2.8	0.852
Location			0.754
Antrum	10 (20.8)	8 (20.5)	
Body	32 (66.7)	28 (71.8)	
Fundus	6 (12.5)	3 (7.7)	

^†^According to Kimura-Takemoto classification in white light endoscopy image.

EMR: endoscopic mucosal resection and ESD: endoscopic submucosal dissection.

**Table 2:** Complete resection rate in endoscopic mucosal resection and endoscopic submucosal dissection groups.

	EMR (*n* = 48)	ESD (*n* = 39)	*P* value
Endoscopic complete resection, *n* (%)	45 (93.7)	38 (97.4)	0.624
Histologic complete resection, *n* (%)	40 (83.3)	37 (94.9)	0.174
Lateral margin involvement, *n* (%)	2 (4.2)	1 (2.6)	>0.999
Vertical margin involvement, *n* (%)	8 (16.7)	1 (2.6)	0.038*

EMR: endoscopic mucosal resection and ESD: endoscopic submucosal dissection; *odd ratio was 7.6.

**Table 3:** Microscopic findings of resected carcinoid tumors.

	EMR (*n* = 48)	ESD (*n* = 39)	*P* value
Lymphovascular findings			
Lymphatic invasion, *n* (%)	1 (2.1)	0 (0.0)	0.138
Vascular invasion, *n* (%)	0 (0.0)	0 (0.0)	NA
Invading to muscular propria, *n* (%)	0 (0.0)	0 (0.0)	NA
Mitotic index			
<2/HPF, *n* (%)	46 (95.8)	37 (94.9)	>0.999
2–20/HPF, *n* (%)	2 (4.1)	2 (5.1)	
Ki 67 index			
<2%, *n* (%)	38 (79.2)	34 (87.2)	0.404
3–20%, *n* (%)	10 (42.7)	5 (12.8)	

EMR: endoscopic mucosal resection, ESD: endoscopic submucosal dissection, and NA: not accessible.

**Table 4:** Complication and procedure duration in endoscopic mucosal resection and endoscopic submucosal dissection groups.

	EMR (*n* = 48)	ESD (*n* = 39)	*P* value
Immediate bleeding, *n* (%)	3 (6.3)	6 (15.4)	0.288
Delayed bleeding, *n* (%)	2 (4.2)	2 (5.1)	>0.999
Perforation, *n* (%)	0 (0.0)	1 (2.6)	0.448
Surgery due to complication, *n* (%)	0 (0.0)	0 (0.0)	NA
Procedure time, mean ± SD, min	26.1 ± 10.5	9.5 ± 3.6	<0.001

EMR: endoscopic mucosal resection, ESD: endoscopic submucosal dissection, and NA: not accessible.

## Abbreviations

EMR: Endoscopic mucosal resection
ESD: Endoscopic submucosal dissection
CT: Computed tomography.
